# Supplementary material for: The Small RNA Universe of Capitella teleta
Source: Front Mol Biosci. 2022 Feb 25;9:802814. doi: 10.3389/fmolb.2022.802814 (PMC8915122; doi:10.3389/fmolb.2022.802814)
Supplement: Supplementary file 1 [file DataSheet1.ZIP › Supplement/confident/CAPTEscaffold_324_18342.pdf]

Provisional ID	:	CAPTEscaffold_324_18342
Score total	:	172.8
Score for star read(s)	:	3.9
Score for read counts	:	168.9
Score for mfe	:	-0.9
Score for randfold	:	1.6
Score for cons. seed	:	-0.6
Total read count	:	343
Mature read count	:	221
Loop read count	:	0
Star read count	:	122

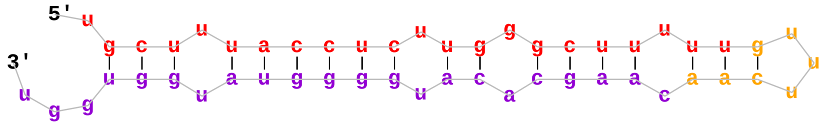

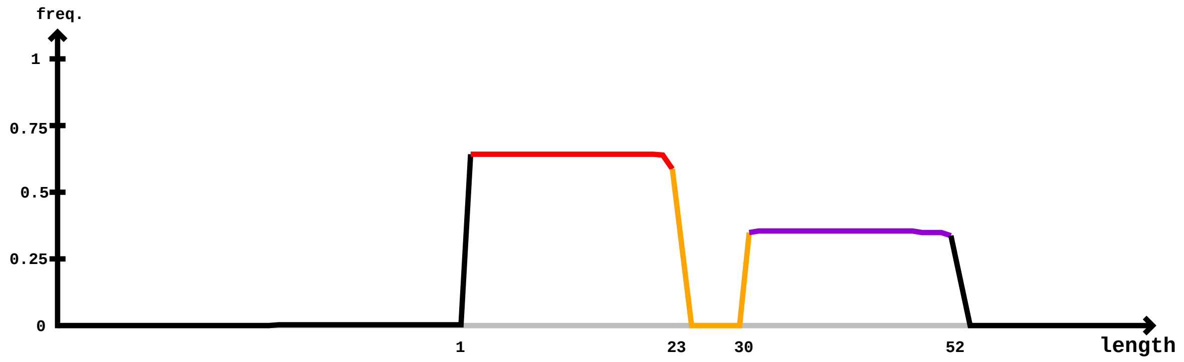

Mature **Star**

```
5'- uccacauuuuaagaugaccaacaauucugcuuggcuuguguugcuuuaccucuugggcuuuuuguuucaacaagcacaugggguaugguggugguaugcccaagucuuugcaa   -3'   obs
    uccacauuuuaagaugaccaacaauucugcuuggcuuguguugcuuuaccucuugggcuuuuuguuucaacaagcacaugggguaugguggugguaugcccaagucuuugcaa         exp
    ....((((....))))...........(((..((((((.((((((.((((((.((.((((.(((...))).)))).)).)))))).))))......)).))))))...))).   reads   mm   sample
    .....................caauucugcuuggcuugugu.......................................................................   1       0    seq
    .........................................ugcuuuaccucuugggcuuu...................................................   1       0    seq
    .........................................ugcuuuaccucuugggcuuuu..................................................   18      0    seq
    .........................................ugcuuuaccucuugggcuuuuu.................................................   150     0    seq
    .........................................ugcuuAaccucuugggcuuuuu.................................................   1       1    seq
    .........................................ugcuuuaccucuugggUuuuuu.................................................   2       1    seq
    .........................................ugcuuuaccucuuAggcuuuuu.................................................   2       1    seq
    .........................................ugcuuuaccucuugggcuGuuu.................................................   12      1    seq
    .........................................ugcuuuaccucuugggcuuuuuU................................................   30      1    seq
    .........................................ugcuuuaccucuugggcuuuuuA................................................   5       1    seq
    .....................................................................caagcacaugggguaugg.........................   2       0    seq
    .....................................................................caagcacaugggguauggugg......................   4       0    seq
    .....................................................................caagcacaugggguauggugG.......................   1       1    seq
    .....................................................................caagcacaugggguaugguAgu.....................   48      1    seq
    .....................................................................caagcacaugggguaugguggu.....................   60      0    seq
    .....................................................................Aaagcacaugggguaugguggu.....................   1       1    seq
    .....................................................................Gaagcacaugggguaugguggu.....................   2       1    seq
    .....................................................................caagcacaugggguauggugguU....................   1       1    seq
    .....................................................................caagcacaugggguauggugguA....................   1       1    seq
    ......................................................................aagcacaugggguauggugguA....................   2       1    seq
```
